# GCNCDA: A new method for predicting circRNA-disease associations based on Graph Convolutional Network Algorithm

**DOI:** 10.1371/journal.pcbi.1007568

**Published:** 2020-05-20

**Authors:** Lei Wang, Zhu-Hong You, Yang-Ming Li, Kai Zheng, Yu-An Huang

**Affiliations:** 1 College of Information Science and Engineering, Zaozhuang University, Zaozhuang, China; 2 Xinjiang Technical Institutes of Physics and Chemistry, Chinese Academy of Sciences, Urumqi, China; 3 Department of Electrical Computer and Telecommunications Engineering Technology, Rochester Institute of Technology, Rochester, United States of America; 4 School of Computer Science and Technology, China University of Mining and Technology, Xuzhou, China; 5 Department of Computing, Hong Kong Polytechnic University, Hong Kong, China; University of Calgary, CANADA

## Abstract

Numerous evidences indicate that Circular RNAs (circRNAs) are widely involved in the occurrence and development of diseases. Identifying the association between circRNAs and diseases plays a crucial role in exploring the pathogenesis of complex diseases and improving the diagnosis and treatment of diseases. However, due to the complex mechanisms between circRNAs and diseases, it is expensive and time-consuming to discover the new circRNA-disease associations by biological experiment. Therefore, there is increasingly urgent need for utilizing the computational methods to predict novel circRNA-disease associations. In this study, we propose a computational method called GCNCDA based on the deep learning Fast learning with Graph Convolutional Networks (FastGCN) algorithm to predict the potential disease-associated circRNAs. Specifically, the method first forms the unified descriptor by fusing disease semantic similarity information, disease and circRNA Gaussian Interaction Profile (GIP) kernel similarity information based on known circRNA-disease associations. The FastGCN algorithm is then used to objectively extract the high-level features contained in the fusion descriptor. Finally, the new circRNA-disease associations are accurately predicted by the Forest by Penalizing Attributes (Forest PA) classifier. The 5-fold cross-validation experiment of GCNCDA achieved 91.2% accuracy with 92.78% sensitivity at the AUC of 90.90% on circR2Disease benchmark dataset. In comparison with different classifier models, feature extraction models and other state-of-the-art methods, GCNCDA shows strong competitiveness. Furthermore, we conducted case study experiments on diseases including breast cancer, glioma and colorectal cancer. The results showed that 16, 15 and 17 of the top 20 candidate circRNAs with the highest prediction scores were respectively confirmed by relevant literature and databases. These results suggest that GCNCDA can effectively predict potential circRNA-disease associations and provide highly credible candidates for biological experiments.

## Author summary

The recognition of circRNA-disease association is the key of disease diagnosis and treatment, and it is of great significance for exploring the pathogenesis of complex diseases. Computational methods can predict the potential disease-related circRNAs quickly and accurately. Based on the hypothesis that circRNA with similar function tends to associate with similar disease, GCNCDA model is proposed to effectively predict the potential association between circRNAs and diseases by combining FastGCN algorithm. The performance of the model was verified by cross-validation experiments, different feature extraction algorithm and classifier models comparison experiments. Furthermore, 16, 15 and 17 of the top 20 candidate circRNAs with the highest prediction scores in disease including breast cancer, glioma and colorectal cancer were respectively confirmed by relevant literature and databases. It is anticipated that GCNCDA model can give priority to the most promising circRNA-disease associations on a large scale to provide reliable candidates for further biological experiments.

## Introduction

As a new type of endogenous non-coding RNA, circular RNA (circRNA) has a closed-loop structure without a 5’and 3’polyadenylated tails [1–3]. As early as 1971, researchers discovered the viroids genome composed of single-stranded closed RNA molecules in potatoes [4]. In 1979, Hsu *et al*. [5] observed the presence of circRNA in the cytoplasm of eukaryotic cells by electron microscopy. In 1995, the researchers [6] found that the mouse sperm determinant gene Sry has circular transcription during transcription. But these findings did not attract much attention of researchers at the time. Until 2012, Salzman *et al*. [7] reported about 80 circRNAs for the first time with the help of high-throughput sequencing technology. Since then, a large number of circRNA molecules have been identified.

With the rapid development of bioinformatics and the continuous innovation of high-throughput sequencing technology, a large number of endogenous circRNA have been found in eukaryotic cells. CircRNA has the characteristics of universality, conservativeness, tissue-specificity and stability. Its unique sequence structure makes it have the functions of microRNA sponge [8], regulators of RNA binding proteins [9] and transcription of parental genes [10]. In addition, it is involved in the development and progression of diseases such as cancer [11, 12], diabetes [13], nervous system diseases [14] and atherosclerosis [15]. For example, Burd *et al*. [16] found that the expression of cANRIL (circular antisense non-coding RNA in the INK4 locus) is an antisense transcript of INK4/ARF gene, which can inhibit the expression of INK4/ARF through specific multi comb family complex, thereby affecting the risk of atherosclerosis. Du *et al*. [17] found that circ-Foxo3, a member of the transcription factor foxo3, is highly expressed in myocardial samples from elderly patients and rats. It can prevent and reposition ID-1, E2F1, FAK and H1F1a in the cytoplasm and prevent their anti-aging function. By establishing the HT22 cell model of oxygen-glucose deprivation/reoxygenation (OGD/R), Lin *et al*. [18] found that the expression of mmu-circRNA-015947 was higher than that of normal cells, indicating that the expression of circRNA was involved in OGD/R-induced neuron injury. Lukiw [19] found that in the hippocampal CA1 region of Alzheimer’s disease (AD), there is a dysregulation of the miRNA-circRNA system. When the expression of CDRlas (CiRS-7) decreased or the ability to adsorb microRNA-7 weakened, the expression of miR-7 is increased and directly leads to down-regulation of ubiquitin ligase an expression in the human central nervous system, thereby affecting the normal function of the central nervous system and causing serious damage to brain tissue. Numerous studies have shown that circRNA can be a new clinical diagnostic marker or a potential target for human disease treatment. Therefore, the identification of disease-related circRNA may help to reveal the mechanism of disease occurrence and development, and further promote the understanding of complex human diseases.

As the number of detected circRNAs increases, multiple databases have been created to store information on circRNAs, such as Circ2Traits [20], circBase [21], deepBase [22] and CircNet [23]. Furthermore, researchers have gradually collected circRNA-disease associations supported by experiments and established databases, such as circR2Disease [24], circRNADb [25], circRNADisease [26] and Circ2Disease [27]. The accumulation of these data provides an opportunity for computational methods to predict potential circRNA-disease associations. For example, Xiao *et al*. [28] proposed an integrated computational framework called MRLDC to identify disease-associated circRNAs based on the hypothesis that circRNAs with similar functions are usually associated with similar diseases, and vice versa. Yan *et al*. [29] developed the DWNN-RLS method using Regularized Least Squares of Kronecker product kernel to predict circRNA-disease associations. In the experiment, this method achieved AUC of 0.8854, 0.9205 and 0.9701 in 5-fold CV, 10-fold CV and LOOCV, respectively. Fan *et al*. [30] proposed the KATZHCDA model for predicting circRNA-disease associations based on a heterogeneous network constructed by disease phenotype similarity, circRNA expression profiles and Gaussian interaction profile kernel similarity. As a result, KATZHCDA reached the AUC values of 0.7936 and 0.8469 in 5-fold cross-validation and LOOCV, respectively. Although the above models play important roles in the development of circRNA-disease association prediction computational methods and have achieved fruitful results, they are limited by certain problems: (1) the existing data are derived from incompletely related biological information, which cannot fully describe the complex association between circRNA and disease. (2) The experimentally verified circRNA-disease associations are limited in number and have some noise information, which easily leads to many false negative associations predicted by the model.

The purpose of this study is to propose a new computational model to predict the potential circRNA-disease associations in an attempt to overcome these problems. The proposed model GCNCDA has the following advantages: (1) Comprehensive use of disease semantic similarity information, disease GIP kernel similarity information, circRNA GIP kernel similarity information and known circRNA-disease association information to accurately predict potential circRNA-disease associations. (2) The advanced features of circRNA-disease associations are extracted by the deep learning FastGCN algorithm to reduce false negative associations and improve model performance. In the 5-fold cross-validation experiment on the benchmark dataset, GCNCDA achieved an AUC value of 90.90%. The results of comparative experiments show that GCNCDA is superior to other competing models and can effectively predict potential circRNA-disease associations. Furthermore, case studies show that GCNCDA can identify new circRNA-disease associations, which are validated by the latest literature and databases. It is worth noting that the performance of GCNCDA is underestimated due to experimentally verified limitations on the number of circRNA-disease associations.

## Results and discussion

### Evaluation criteria

In this study, we used the 5-fold cross-validation (5-fold CV) method to evaluate the performance of the model. This method can not only reduce over-fitting to a certain extent but also obtain as much effective information as possible from limited data [31]. More concretely, we first randomly divide the initial dataset into five sub-data sets. When the method is executed, a separate sub-data set is reserved for validating the model and the other four sub-data sets are used to train the model. This process is repeated 5 times until each sub-data set is verified once and only verified once. Finally, the average results of these 5 times are used as the performance indicators of the model. General evaluation criteria are used in this study to evaluate the performance of GCNCDA, including accuracy (Accu.), Sensitivity (Sen.), precision (Prec.), F1-Score (F1) and Matthews Correlation Coefficient (MCC). They are defined as:

$$Accu.=\frac{TP+TN}{TP+TN+FP+FN}$$ (1)

$$Sen.=\frac{TP}{TP+FN}$$ (2)

$$Prec.=\frac{TP}{TP+FP}$$ (3)

$$F1=\frac{2TP}{2TP+FP+FN}$$ (4)

$$MCC=\frac{TP\times TN-FP\times FN}{\sqrt{\left(TP+FP)(TP+FN)(TN+FP)(TN+FN\right)}}$$ (5)

Here, TP means true positive, TN means true negative, FP means false positive, and FN means false negative. Furthermore, we also plot the Receiver Operating Characteristic (ROC) [32, 33] curves of the 5-fold CV generated by GCNCDA and calculate their average area under the ROC curve (AUC) [34].

### Model performance evaluation

**Fig 1:**
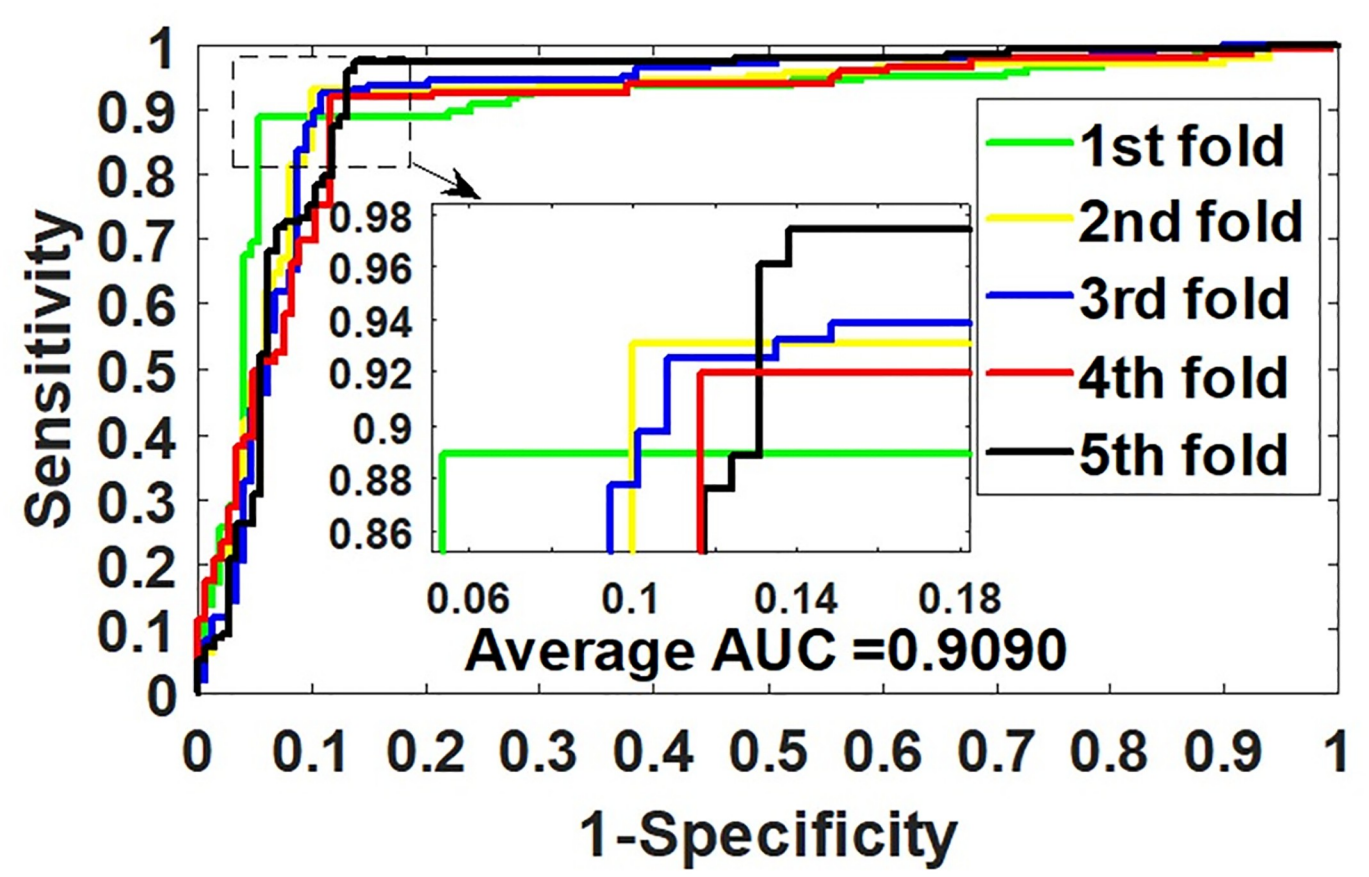
ROC curves of 5-fold CV obtained by GCNCDA on circR2Disease dataset.

In the experiment, GCNCDA is implemented on the benchmark dataset circR2Disease to evaluate its ability to predict potential circRNA-disease associations. The detailed results of 5-fold CV are summarized in Table 1. As can be seen from the table, GCNCDA achieved an average accuracy of 91.20% and a standard deviation of 0.74%, of which the accuracy of 5-fold experiments was 91.86%, 91.19%, 90.85%, 90.17% and 91.95%, respectively. In terms of accuracy, sensitivity, precision, F1-Score, Matthews correlation coefficient and area under ROC curve, GCNCDA obtained 92.78%, 90.03%, 91.33%, 82.55% and 90.90%, with standard deviations of 3.03%, 2.37%, 0.78%, 1.60% and 0.81%, respectively. Fig 1 plots the ROC curve generated by GCNCDA using 5-fold CV on the circR2Disease dataset. From the experimental results, we can observe that GCNCDA performs well and can effectively predict the potential disease-related circRNAs.

**Table 1:** Results of 5-fold CV generated by GCNCDA on circR2Disease dataset.

Test set	Accu. (%)	Sen. (%)	Prec. (%)	F1 (%)	MCC (%)	AUC (%)
1	91.86	88.97	94.16	91.49	83.83	90.93
2	91.19	93.10	89.40	91.22	82.45	90.54
3	90.85	92.52	89.47	90.97	81.74	91.24
4	90.17	91.95	88.96	90.43	80.38	89.80
5	91.95	97.39	88.17	92.55	84.33	92.00
Average	**91.20±0.74**	**92.78±3.03**	**90.03±2.37**	**91.33±0.78**	**82.55±1.60**	**90.90±0.81**

### Comparison of different classifier models

**Fig 2:**
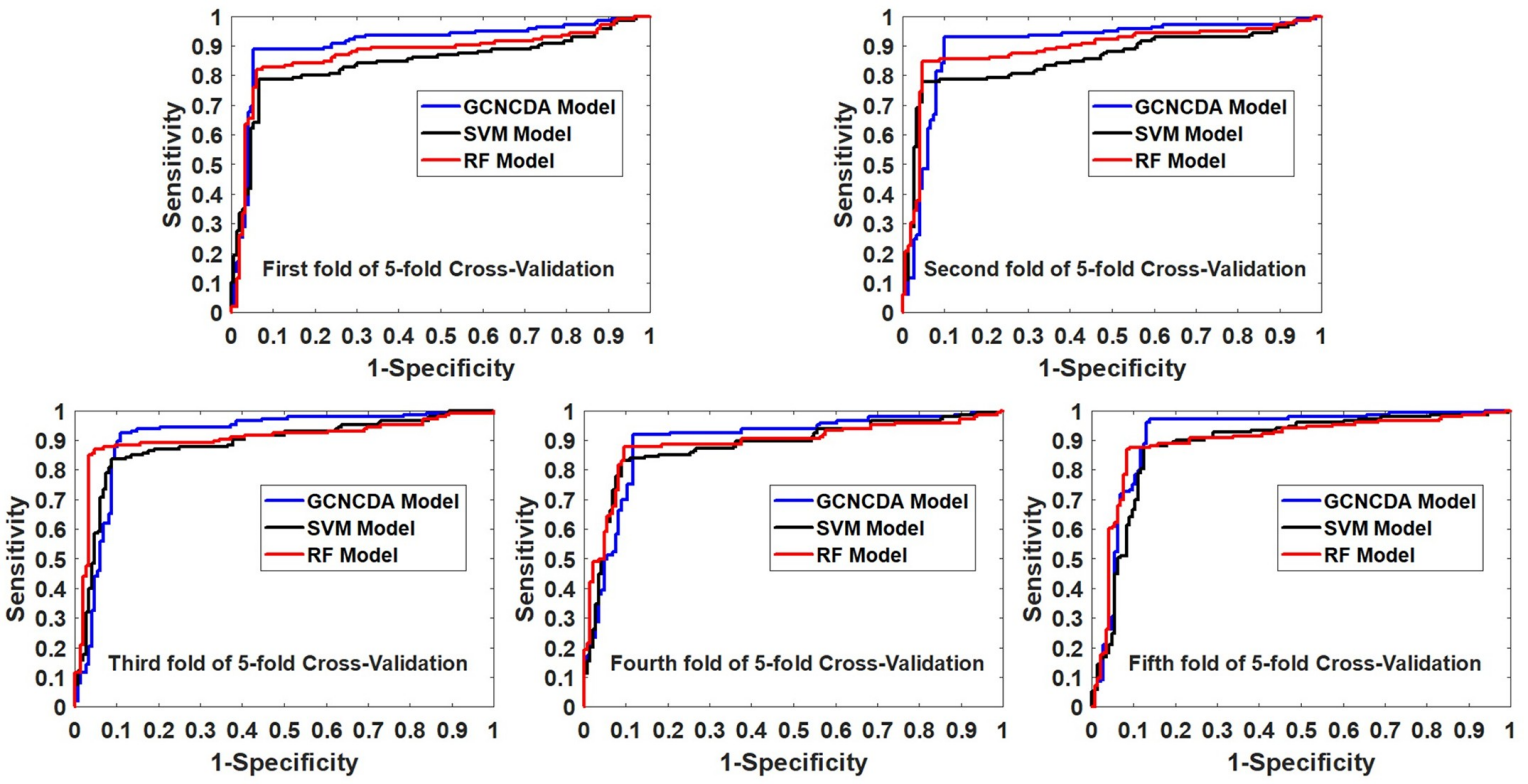
Comparison of ROC curves obtained by different classifier models in 5-fold CV on circR2Disease dataset.

**Fig 3:**
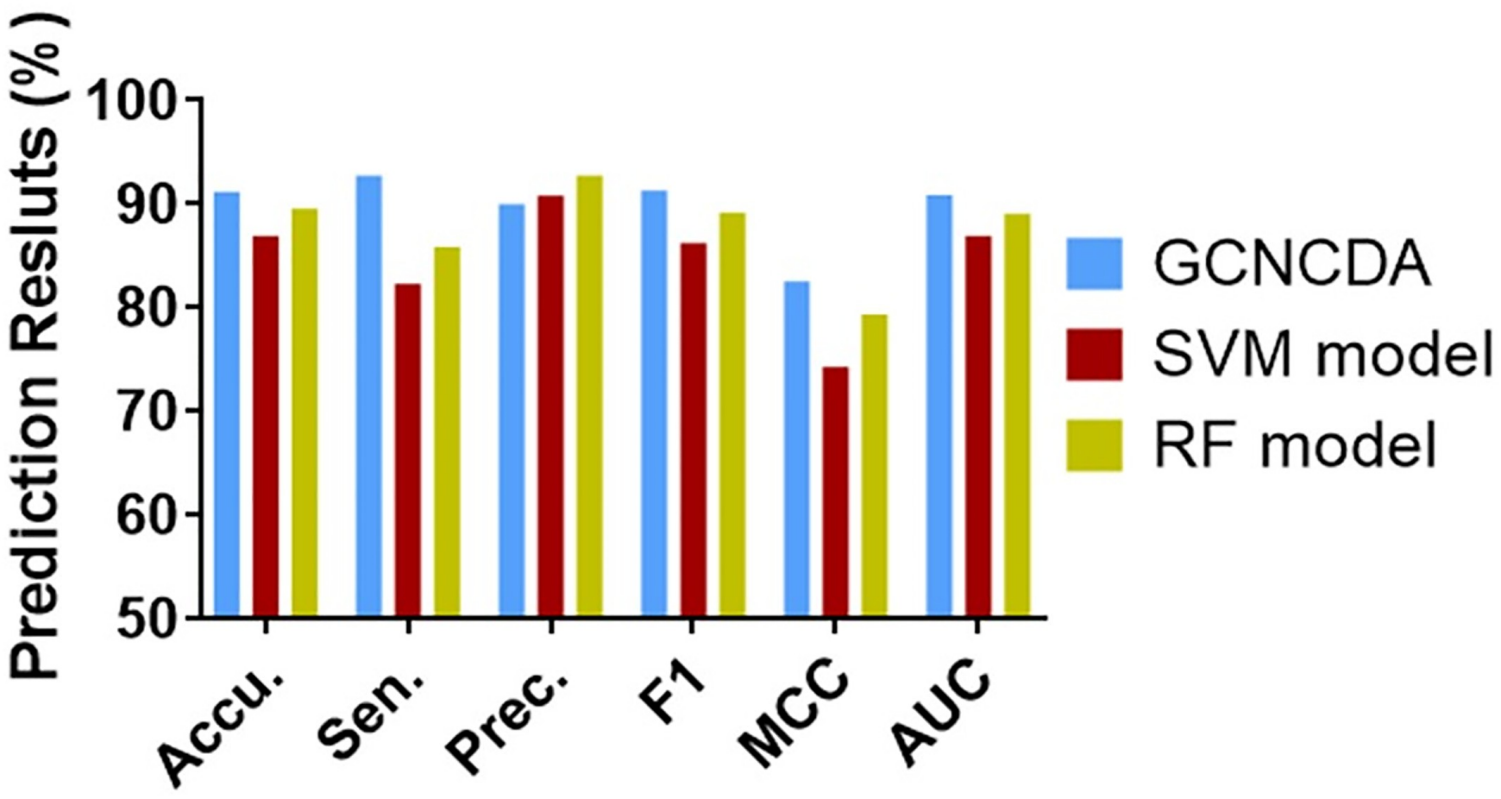
Comparison of results of different classifier models on circR2Disease dataset.

To evaluate the impact of the Forest PA classifier on the overall performance of GCNCDA, we compared different classifier models in this experiment. Specifically, when constructing different classifier models, we keep the other parts of the model unchanged, including the composition of descriptors and feature extraction, and only replace the Forest PA classifier with state-of-the-art Support Vector Machine (SVM) and Random Forest (RF) classifiers, respectively. The SVM model and the RF model are thus constructed and implemented on the circR2Disease dataset using 5-fold CV. Table 2 lists the results of the 5-fold CV experiments performed by these two models. Fig 2 shows a comparison of 5-fold CV ROC curves of different classifier models on the circR2Disease dataset. For the convenience of visual comparison, we display these results in the form of a histogram. As can be seen from Fig 3, GCNCDA achieved the best results in accuracy, sensitivity, F1, MCC and AUC, and achieved the third result in precision, but only 2.75% lower than the best result. From the overall performance point of view, GCNCDA is better than SVM model and RF model. This result indicates that the Forest PA classifier is suitable for GCNCDA model and contributes to the improvement of the model performance.

**Table 2:** Results of 5-fold CV generated by SVM model and RF model on circR2Disease dataset.

Test set	Accu. (%)	Sen. (%)	Prec. (%)	F1 (%)	MCC (%)	AUC (%)
1	86.10	78.62	91.94	84.76	72.87	84.30
2	86.78	77.93	94.17	85.28	74.56	85.54
3	87.46	83.67	90.44	86.93	75.12	88.49
4	87.12	83.22	90.51	86.71	74.50	87.96
5	87.25	88.24	87.10	87.66	74.48	88.50
**SVM Model**	**86.94±0.53**	**82.34±4.20**	**90.83±2.58**	**86.27±1.21**	**74.31±0.84**	**86.96±1.92**
1	88.14	82.07	92.97	87.18	76.73	87.37
2	90.17	84.83	94.62	89.45	80.72	89.08
3	91.19	87.07	94.81	90.78	82.64	90.41
4	89.15	87.92	90.34	89.12	78.34	88.85
5	89.26	87.58	91.16	89.33	78.59	89.54
**RF Model**	**89.58±1.15**	**85.89±2.46**	**92.78±2.01**	**89.17±1.29**	**79.41±2.30**	**89.05±1.11**
**GCNCDA**	**91.20±0.74**	**92.78±3.03**	**90.03±2.37**	**91.33±0.78**	**82.55±1.60**	**90.90±0.81**

### Comparison of different feature extraction algorithms

**Fig 4:**
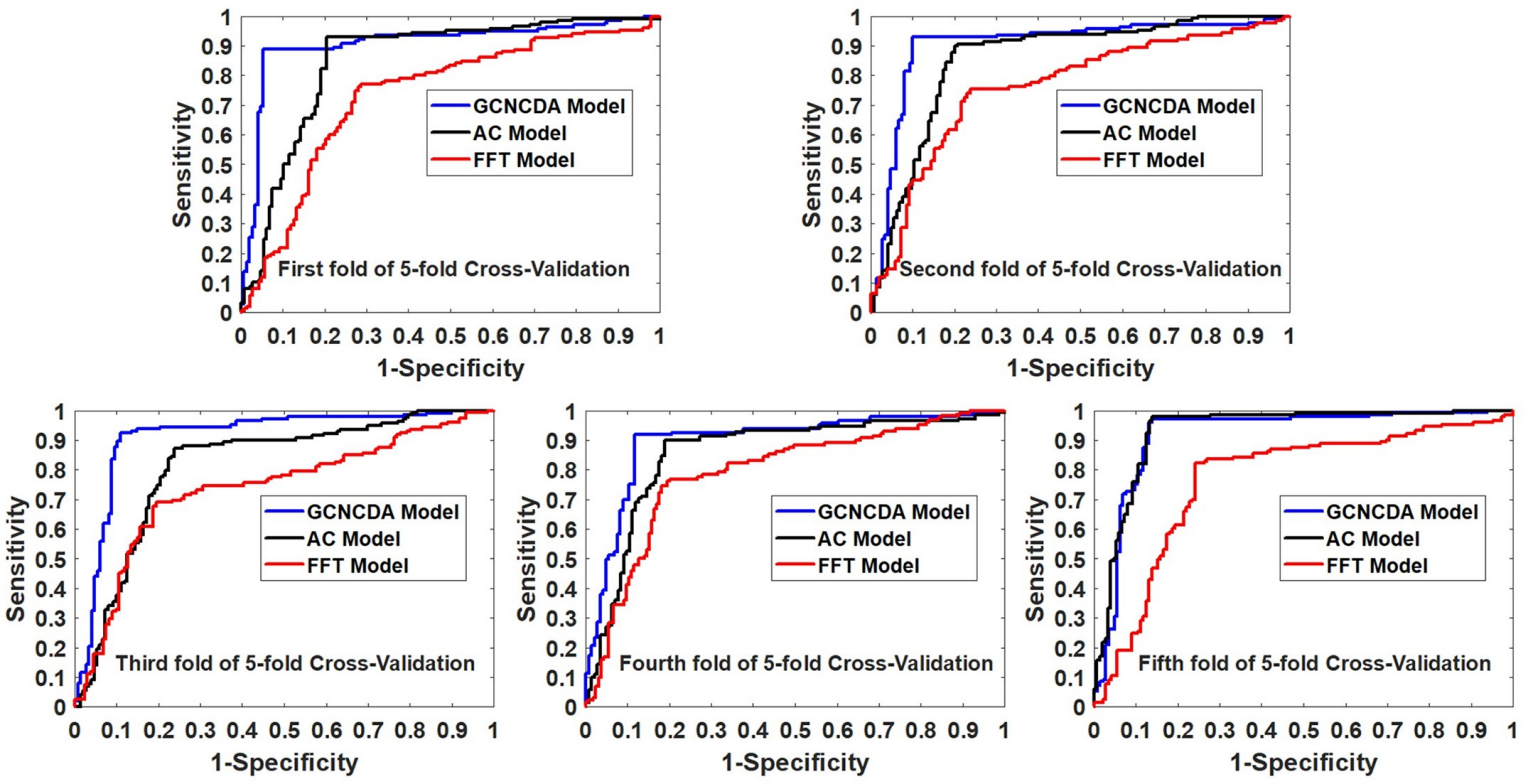
Comparison of ROC curves obtained by different feature extraction models in 5-fold CV on circR2Disease.

**Fig 5:**
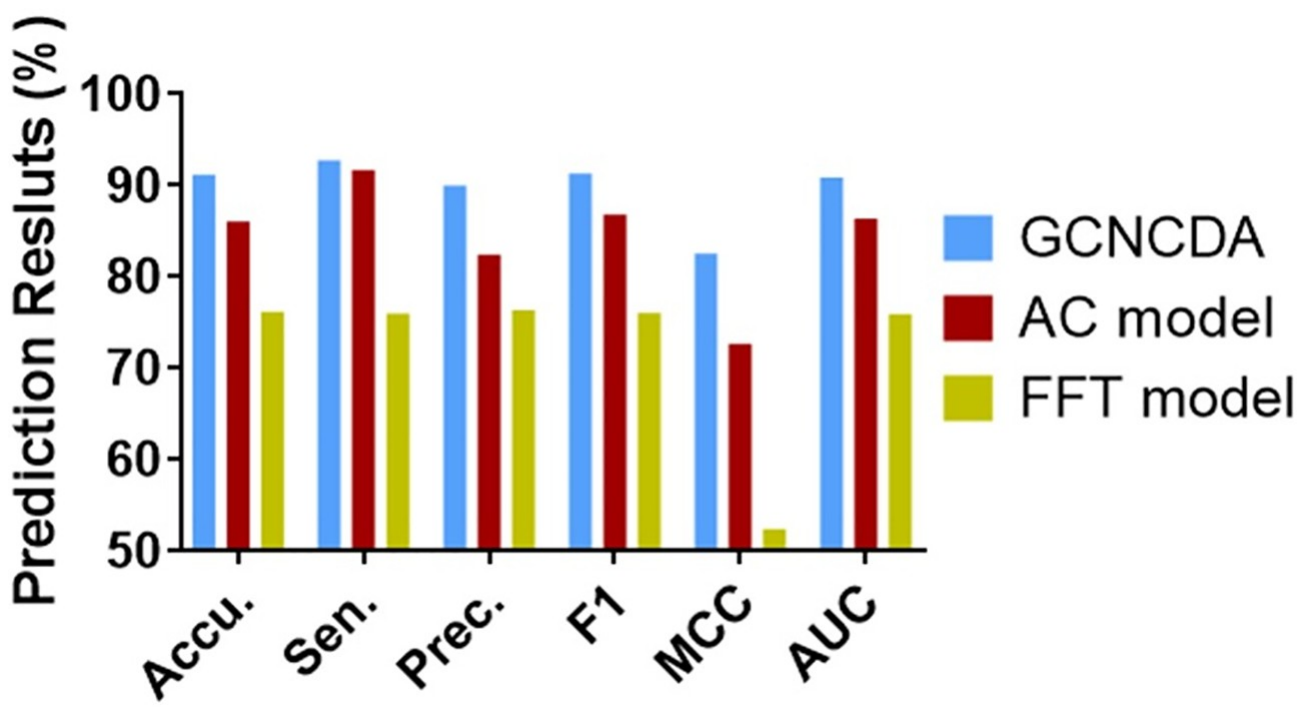
Comparison of results of different feature extraction models on circR2Disease dataset.

In order to evaluate the effect of the FastGCN feature extraction algorithm on the overall performance of GCNCDA, we compared different feature extraction algorithm models in this experiment. Similar to the experiment with different classifiers, when we construct different feature extraction algorithm models, the other parts of the model are unchanged, including the composition of the descriptors and classifier. Only the Auto Covariance (AC) [35] and fast Fourier transform (FFT) [36] extraction algorithms are used instead of the FastGCN algorithm. The AC model and the FFT model are thus constructed and implemented on the circR2Disease dataset using 5-fold CV. Table 3 summarizes the results of the 5-fold CV obtained by the two models. Fig 4 shows a comparison of 5-fold CV ROC curves of different feature extraction models on the circR2Disease dataset. Similarly, we used a histogram to visually compare the results of the three models. As can be seen from Fig 5, GCNCDA achieved the best results in all the evaluation criteria, including accuracy, sensitivity, precision, F1, MCC and AUC. The experimental results show that the FastGCN algorithm can effectively extract the advanced features of the fusion descriptor, thus helping to improve the performance of the model. In addition, from the comparison experiments of different classifiers and extraction algorithms, we can also see that the FastGCN algorithm is more helpful to the performance improvement of the model than the Forest PA classifier. This suggests that the FastGCN algorithm is the key to the GCNCDA model and plays an important role in predicting potential disease-associated circRNAs.

**Table 3:** Results of 5-fold CV generated by AC model and FFT model on circR2Disease dataset.

Test set	Accu. (%)	Sen. (%)	Prec. (%)	F1 (%)	MCC (%)	AUC (%)
1	86.44	93.24	82.14	87.34	73.55	85.39
2	85.08	90.67	81.93	86.08	70.52	85.83
3	81.36	86.71	77.50	81.85	63.23	81.78
4	85.76	90.13	83.54	86.71	71.67	85.74
5	91.95	97.95	87.20	92.26	84.54	93.16
**ACModel**	**86.12±3.81**	**91.74±4.18**	**82.46±3.48**	**86.85±3.71**	**72.70±7.69**	**86.38±4.15**
1	73.90	76.32	73.89	75.08	47.72	73.59
2	75.93	75.52	75.00	75.26	51.83	76.38
3	74.24	68.94	81.02	74.50	49.46	73.96
4	78.64	76.92	75.19	76.05	56.80	79.21
5	78.19	82.35	76.83	79.50	56.41	76.74
**FFT Model**	**76.18±2.19**	**76.01±4.78**	**76.38±2.80**	**76.08±1.99**	**52.44±4.07**	**75.98±2.29**
**GCNCDA**	**91.20±0.74**	**92.78±3.03**	**90.03±2.37**	**91.33±0.78**	**82.55±1.60**	**90.90±0.81**

### Comparison with other existing methods

At present, some researchers have established models for predicting circRNA-disease associations based on the benchmark dataset circR2Disease, including DWNN-RLS [29], KATZHCDA [30], PWCDA [37], GHICD [37] and RWRHCD [37]. To evaluate the performance of GCNCDA, we compared it to the 5-fold CV AUC results of these models. Table 4 summarizes the 5-fold CV AUC scores generated by the various models on the same benchmark dataset circR2Disease. From the table we can see that GCNCDA is outperforms other existing methods. This indicates that the GCNCDA model, which uses the FastGCN algorithm to extract circRNA and disease fusion information features and combines the Forest PA classifier, can effectively improve the predictive performance of circRNA-disease associations.

**Table 4:** The 5-fold CV AUC scores generated by the various models on the same benchmark dataset circR2Disease.

Methods	GCNCDA	DWNN-RLS	KATZHCDA	PWCDA	GHICD	RWRHCD
AUC	90.90	88.54	79.36	89.00	72.90	66.60

### Case studies

To demonstrate the capability of GCNCDA to predict new disease-associated circRNAs based on known circRNA-disease associations, the performance of GCNCDA was further evaluated. Specifically, all known circRNA-disease associations in benchmark dataset $R^{+}$ were used to train GCNCDA, and the remaining unknown circRNA-disease associations were considered candidates for testing. All candidates were then ranked based on GCNCDA predictive scores in diseases including Breast Cancer, Glioma, and Colorectal Cancer. Finally, the predicted disease-circRNA associations were confirmed by searching the latest published literature and circRNA-disease databases.

Breast cancer is one of the most common malignant tumors in the world, and its incidence has been increasing since the late 1970s. There is increasing evidence that circRNAs can be used as effective biomarkers for the diagnosis of breast cancer. Therefore, we chose breast cancer for testing to verify the predictive ability of GCNCDA. The prediction results are shown in Table 5, from which we can see that 16 of the top 20 candidates with the highest prediction scores were confirmed by relevant literature and datasets. For example, the hsa_circ_0007534 with the highest prediction score was confirmed by Zhou *et al*. [38], which can suppresses the migration and invasion of breast cancer cells line MCF-7 by down-regulating targeting RFC3.

**Table 5:** The top 20 breast cancer related candidate circRNAs.

Breast Cancer					
Rank	circRNA	Evidence	Rank	circRNA	Evidence
1	hsa_circ_0007534	PMID:29593432	11	hsa_circ_0006528	circRNAdisease
2	circHIPK3/hsa_circ_0000284	PMID:27050392	12	hsa_circ_0001785	unconfirmed
3	hsa_circ_0001982	circRNAdisease	13	circGFRA1/hsa_circ_005239	PMID:29037220
4	circPVT1/hsa_circ_0001821	PMID:27928058	14	hsa_circ_0002874	circRNAdisease
5	hsa_circ_0093859	PMID:29593432	15	circMED13	PMID:29221160
6	hsa_circ_0092276	circRNAdisease	16	hsa_circ_0047905	unconfirmed
7	hsa_circ_0001313/circCCDC66	PMID:28249903	17	hsa_circ_0085495	circRNAdisease
8	hsa_circ_0108942	circRNAdisease	18	hsa_circ_0043256	unconfirmed
9	hsa_circ_0003838	circRNAdisease	19	hsa_circ_0005402	unconfirmed
10	circFoxo3/hsa_circ_0006404	PMID:26657152	20	circDENND4C	PMID:28739726

Glioma is one of the most common primary intracranial tumors, accounting for approximately 30% of all brain tumors and central nervous system tumors, and 80% of all malignant brain tumors. Table 6 lists the top 20 glioma related candidate circRNAs predicted by GCNCDA with the highest scores, 15 of which were confirmed by relevant literature and datasets. For example, Barbagallo *et al*. [39] identified CDR1-AS as the downstream target of miR-671-5p in human glioblastoma multiforme (GBM) by combining in silico and in vitro approach, which participated in the biopathological changes of GBM cells. This result is consistent with our prediction of the candidate with the second highest score.

**Table 6:** The top 20 glioma related candidate circRNAs.

Glioma					
Rank	circRNA	Evidence	Rank	circRNA	Evidence
1	hsa_circ_0004214	PMID:28622299	11	hsa_circ_0008717	unconfirmed
2	CDR1-AS	PMID:26683098	12	circ_FKBP8	circRNADisease
3	circ_COL1A2	circRNADisease	13	hsa_circ_0000177	circFunbase
4	circ_SPTAN1	circRNADisease	14	hsa_circ_0007385	unconfirmed
5	circETFA	PMID:26873924	15	hsa_circ_0000284/circHIPK3	PMID:30057315
6	hsa_circ_0015758	circFunbase	16	hsa_circ_0024108	unconfirmed
7	cir-ITCH/hsa_circ_0001141	PMID:29887952	17	hsa_circ_0001649	PMID:29343848
8	circ_RIMS1	circRNADisease	18	circ_SMARCA5	PMID:26873924
9	hsa_circ_0000936	circFunbase	19	hsa_circ_0051172	unconfirmed
10	circ_ZNF148	PMID:26873924	20	hsa_circ_0001982	unconfirmed

Colorectal cancer is one of the common types of cancer in women, and its morbidity and mortality are among the highest in the world. According to statistics, colorectal cancer patients are widely distributed, especially in economically developed regions. We summarize in Table 7 the top 20 circRNAs predicted by the GCNCDA with the highest scores related to colorectal cancer, of which 17 were confirmed by relevant literature and datasets. For example, circ-KLDHC10 with the highest predicted score was confirmed by Yan *et al*. [40], and its expression level in cancer serum was significantly higher than that in the normal control group, which indicates that circ-KLDHC10 is enriched and stable in exosomes and can be a promising biomarker for cancer diagnosis.

**Table 7:** The top 20 colorectal cancer related candidate circRNAs.

Colorectal Cancer					
Rank	circRNA	Evidence	Rank	circRNA	Evidence
1	circ-KLDHC10	PMID:26138677	11	hsa_circ_0014717	PMID:29571246
2	hsa_circ_0020397	circRNADisease	12	hsa_circ_0007534	PMID:29364478
3	hsa_circ_0000504	circRNADisease	13	hsa_circ_0003707	unconfirmed
4	hsa_circ_0001649	PMID:29421663	14	hsa_circ_0000284	PMID:27050392
5	has-circ_0006174	circRNADisease	15	hsa_circ_0048232	circRNADisease
6	hsa_circ_0074930	circRNADisease	16	hsa_circrna_104700	circRNADisease
7	circ_HIPK3	PMID:29549306	17	hsa_circ_0007031	unconfirmed
8	hsa_circ_0000069	circRNADisease	18	circ-ZNF609/hsa_circ_0000069	PMID:30570857
9	hsa_circ_0084021	circRNADisease	19	hsa_circ_0008797	unconfirmed
10	hsa_circrna_103809	circRNADisease	20	hsa_circ_0000567	PMID:29333615

## Materials and methods

### Method overview

**Fig 6:**
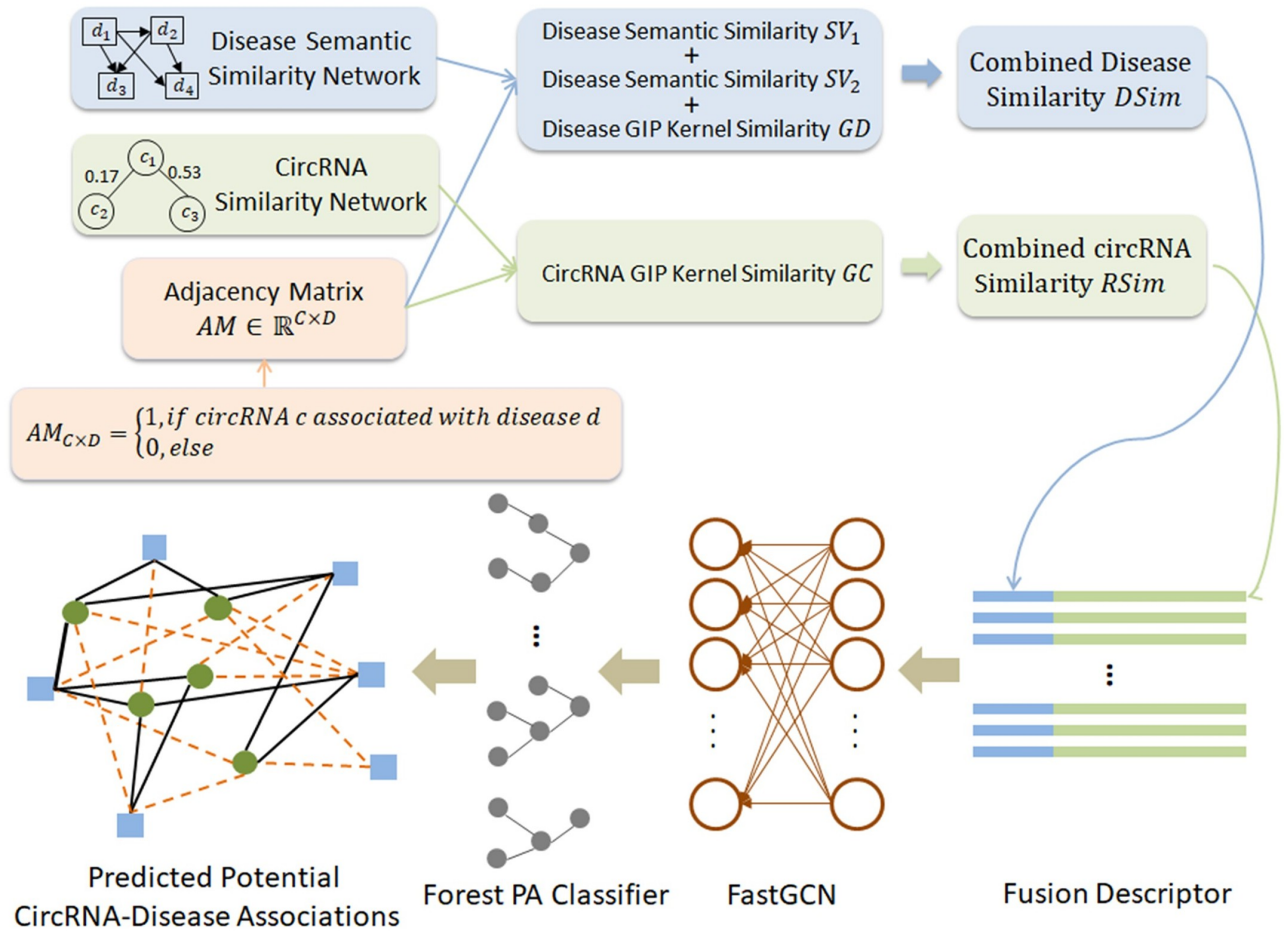
The framework of GCNCDA to predict potential circRNA-disease associations.

In this study, we propose a computational method called GCNCDA to predict potential circRNA-disease associations. The execution process of GCNCDA is divided into the following steps, and its framework is shown in Fig 6. Specifically, we first construct the disease semantic similarity matrix and disease Gaussian interaction profile (GIP) similarity matrix according to disease semantic similarity network and circRNA-disease adjacency matrix. Then, according to circRNA similarity network and circRNA-disease adjacency matrix, construct the circRNA GIP similarity matrix. Next, the disease similarity matrix and circRNA similarity matrix are fused by the fusion strategy to get a unified numerical descriptor. In the fourth step, we use the FastGCN algorithm of deep learning to effectively extract the high-level features of the fusion data and generate the most expressive descriptor. Finally, we feed the extracted high-level features into Forest PA classifier to accurately predict the potential association between circRNAs and diseases. From the execution process of GCNCDA, we can see that the computational resources of model are mainly consumed in the feature extraction stage using FastGCN, so the overall computational complexity of the GCNCDA is O(*N*^3^).

### Benchmark dataset

In this study, we used the recently established experimentally verified circRNA-disease association dataset circR2Disease [24] as the benchmark dataset to evaluate the performance of various models. CircR2Disease is a dedicated database and comprehensive platform that collects disease-related circRNAs from experimental support. The database currently hosts 739 entries from published literature, including 661 circRNAs, and 100 diseases. The benchmark dataset can be expressed as:

$$R=R^{+}\cup R^{-}$$ (6)

where ∪ denotes the union symbol in set theory, $R^{+}$ represents the positive dataset, which contains 739 circRNA-disease associations with experimentally verified, $R^{-}$ represents the negative dataset, which contains 739 circRNA-disease associations without experimentally verified. The circR2Disease dataset can be available on the website http://bioinfo.snnu.edu.cn/CircR2Disease/.

In the circR2Disease dataset, there were a total of 661 × 100 − 739 = 65361 circRNA-disease associations without experimental verified. If they are all treated as negative samples, they will form an unbalanced dataset. In order to avoid bias in the prediction results caused by unbalanced data, we solve this problem by reducing the number of negative samples by the down-sampling method. Specifically, we select 739 negative samples from all negative samples using random sampling without replacement, and then combine the positive samples to form a distributed equilibrium dataset. In theory, there may be unconfirmed circRNA-disease associations in these 65361 negative samples. But in the 739 negative samples we selected, this probability is much less than 739 ÷ (661 × 100 − 739) ≈ 1.13%. Thus, we constructed the dataset containing 1478 samples in this way, in which the number of positive samples is the same as that of negative samples. Known circRNA-disease associations and their names obtatined from circR2Disease database can be seen in Supplementary S1–S3 Tables. The source code and data of GCNCDA model have been uploaded to https://github.com/look0012/GCNCDA/ for researchers to download and use.

Based on the circR2Disease dataset, we constructed 661 × 100 dimensional adjacency matrix *AM*, where 661 represents the number of circRNAs, and 100 represents the number of diseases. When circRNA *c*(*i*) is associated with disease *d*(*i*), element *AM*(*i*, *j*) of matrix *AM* is assigned a value of 1. Otherwise, it is assigned a value of 0.

### Construction of CircRNA similarity model

In this study, we used the Gaussian interaction profile (GIP) kernel similarity to construct the similarity model of circRNA. Based on the hypothesis that circRNAs with similar function are often associated with similar diseases, and vice versa, we established the GIP kernel similarity model of circRNA according to the known circRNA-disease association network. Specifically, we define the binary vector *V*(*c*(*i*)) to represent the interaction profiles of circRNA *c*(*i*). The dimension of the vector *V*(*c*(*i*)) is 100, which corresponds to 100 diseases in adjacent matrix *AM*. When circRNA *c*(*i*) is associated with one of 100 diseases, the corresponding bit in vector *V*(*c*(*i*)) is set to 1. Otherwise, it is set to 0. That is to say, the interaction profiles binary vector *V*(*c*(*i*)) is the row vector of the row corresponding to circRNA *c*(*i*) in the adjacency matrix *AM*. Thus, we can get the circRNA GIP kernel similarity *GC*(*c*(*i*), *c*(*j*)) of circRNA *c*(*i*) and circRNA *c*(*j*):

$$GC(c(i),c(j))=\text{exp}(-\theta_{c}{\left\|V(c(i))-V(c(j))\right\|}^{2})$$ (7)

where *θ*_*c*_ is the width parameter, which can be calculated using the normalized original parameters of the following formula:

$$\theta_{c}=\frac{1}{n}\sum_{i=1}^{n}{\left\|V(c(i))\right\|}^{2}$$ (8)

where *n* is the column number of adjacent matrix *AM*.

### Construction of disease similarity model

The disease similarity model consists of two parts: the disease GIP kernel similarity and the disease semantic similarity. For the disease GIP kernel similarity, our construction method is similar to the GIP kernel similarity of circRNA. More concretely, we define a binary vector *V*(*d*(*i*)) to represent the interaction profiles of disease *d*(*i*) according to the adjacent matrix *AM* provided by circR2Disease dataset. The dimension of the vector *V*(*d*(*i*)) is 661, which corresponds to 661 circRNAs in adjacent matrix *AM*. When disease *d*(*i*) is associated with one of 661 circRNAs, the corresponding bit in vector *V*(*d*(*i*)) is set to 1. Otherwise, it is set to 0. That is to say, the interaction profiles binary vector *V*(*d*(*i*)) is the column vector of the column corresponding to disease *d*(*i*) in the adjacency matrix *AM*. Through the above definition, we can calculate the disease GIP kernel similarity *GD*(*d*(*i*), *d*(*j*)) of disease *d*(*i*) and disease *d*(*j*):

$$GD(d(i),d(j))=\text{exp}(-\theta_{d}{\left\|V(d(i))-V(d(j))\right\|}^{2})$$ (9)

$$\theta_{d}=\frac{1}{m}\sum_{i=1}^{m}{\left\|V(d(i))\right\|}^{2}$$ (10)

where *θ*_*d*_ is the width parameter and *m* is the row number of adjacent matrix *AM*.

For disease semantic similarity, we construct it through the MeSH database [41–43] from the National Library of Medicine (NLM). It can be downloaded at https://www.nlm.nih.gov/. The MeSH database gives a rigorous disease classification system that uses a Directed Acyclic Graph (DAG) to reflect relationships between different diseases. The MeSH dataset can be seen in Supplementary S4 Table. In DAG, a node represents disease, and an edge represents the relationship between diseases. Given a disease *d* whose structure can be expressed as *DAG*_*d*_ = (*d*, *N*_*d*_, *E*_*d*_), where *N*_*d*_ represents the set of diseases associated with *d* including disease *d* itself, and *E*_*d*_ represents the relationship between these diseases. For a disease *s* within *DAG*_*d*_, its contribution value *D*_*d*_(*s*) can be calculated by the following formula:

$$\{\begin{array}{ll} D_{d}\left(s\right)=1 & if\;s=d\\ D_{d}\left(s\right)=\text{max}\left\{\mu \cdot D_{d}\left(s^{\prime }\right)|s^{\prime }\in children\;of\;s\right\} & if\;s\neq d \end{array}$$ (11)

where *μ* indicates the semantic contribution factor between disease *s* and its child disease *s*′. According to the previous study by Wang *et al*. [44], we set the semantic contribution factor *μ* to the optimal value of 0.5. Thus, by accumulating the contribution values of all children with disease *d*, we can get their semantic values *DV*(*d*):

$$DV(d)=\sum_{s\in N_{d}}D_{d}(s)$$ (12)

In general, the more nodes that are shared between DAGs of different diseases, the more similar they are. Based on this assumption, we construct the first disease semantic similarity model *SV*_1_(*d*(*i*), *d*(*j*)) of disease *d*(*i*) and disease *d*(*j*) through the DAG hierarchical relationship of disease:

$${SV}_{1}(d(i),d(j))=\frac{\sum_{s\in N_{d(i)}\cap N_{d(j)}}\left(D_{d(i)}(s)+D_{d(j)}(s)\right)}{DV(d(i))+DV(d(j))}$$ (13)

In disease semantic similarity model *SV*_1_, we mainly consider the hierarchical relationship of disease DAG, that is, the disease in the same layer in the DAG contributes the same value to the disease *d*. However, the number of different diseases in DAGs can also affect the semantic similarity of disease. The fewer diseases appear in DAGs, the more important they are. Therefore, we constructed the second method for calculating the disease contribution value based on this hypothesis:

$$D_{d}^{\prime }(s)=-\text{log}(\frac{num(DAGs(s))}{num(diseases)})$$ (14)

where *num*(*DAGs*(*s*)) denotes the number of DAGs that contain disease *s*, and *num*(*diseases*) denotes the number of all diseases. Thus, the second disease semantic similarity model *SV*_2_(*d*(*i*), *d*(*j*)) of disease *d*(*i*) and disease *d*(*j*) can be calculated as follows:

$${SV}_{2}(d(i),d(j))=\frac{\sum_{s\in N_{d(i)}\cap N_{d(j)}}\left(D_{d(i)}^{\prime }(s)+D_{d(j)}^{\prime }(s)\right)}{DV(d(i))+DV(d(j))}$$ (15)

where *DV*(*d*(*i*)) and *DV*(*d*(*j*)) have the same meaning as disease semantic similarity model *SV*_1_, which can be calculated from formula 7.

### Multi-source data fusion

In order to make full use of information from different sources, we used the fusion method to fuse circRNA similarity information and disease similarity information with known circRNA-disease associations. The fused information can absorb the characteristics of different data sources, thus describing the complex relationship between circRNAs and diseases more comprehensively.

For the circRNA, we use the constructed circRNA GIP kernel similarity *GR* directly to represent the circRNA descriptor *RSim*. For the disease, we need to fuse the disease semantic similarity model *SV*_1_ and *SV*_2_, and disease GIP kernel similarity *GD*. Since the MeSH database provides a strict disease association, we use it as much as possible. More specifically, if there is the semantic similarity between disease *d*(*i*) and disease *d*(*j*), then the disease semantic similarity is used to construct the descriptor *DSim*. Otherwise, it is constructed using disease GIP kernel similarity. This construction rule can be described by the following formula:

$$DSim(d(i),d(j))=\{\begin{array}{cc} \frac{{SV}_{1}(d(i),d(j))+{SV}_{2}(d(i),d(j))}{2} & if\;d(i)\;and\;d(j)\;has\;semantic\;similarity\\ GD(d(i),d(j)) & otherwise \end{array}$$ (16)

Finally, we match circRNA similarity *RSim* with disease similarity *DSim* based on known circRNA-disease associations to form a complete fusion descriptor. The fusion descriptor *FV*(*c*(*i*), *d*(*j*)) of circRNA *c*(*i*) and disease *d*(*j*) can be described as follows:

FV(c(i),d(j))=[RSim(i),DSim(j)] (17)

where *RSim*(*i*) indicates the *i* row vector of circRNA *c*(*i*) in the circRNA similarity matrix *RSim*, and *DSim*(*j*) indicates the *j* column vector of disease *d*(*j*) in the disease similarity matrix *DSim*.

### Feature extraction by fast learning with Graph Convolutional Networks

After getting the fusion descriptors, we used the Fast learning with Graph Convolutional Networks (FastGCN) algorithm to extract their features to remove noise information and improve the performance of the model. FastGCN is an efficient algorithm based on the original GCN and realized by importance sampling. It interprets graph convolutions as integral transforms of embedding functions under probability measure. To be specific, FastGCN interprets the graph vertices as independent and identically distributed (i.i.d.) samples of some probability distributions, and integrates loss and each convolution layer as vertex embedding functions. The integrals are then calculated by Monte Carlo approximation to determine the sample loss and sample gradient. Finally, important sampling is used to reduce the approximate variance. FastGCN not only eliminates the reliance on test data but also produces a controllable cost for each batch of computation.

Suppose there is a graph *G*′ with the vertex set *V*′ associated with a probability space (*V*′, *F*, *P*). For the given graph *G*, it is a subgraph of *G*′ whose vertices are i.i.d. samples of *V*′ obtained from the probability measure *P*. For the probability space, *V*′ is used as the sample space, and *F* can be any event space. The probability measure *P* defines a sample distribution. Thus, the function generalization can be expressed as:

$${\tilde{h}}^{\left(l+1\right)}(v)=\int \hat{A}(v,u)h^{\left(l\right)}(u)W^{\left(l\right)}dP(u),\;h^{\left(l+1\right)}(v)=\sigma ({\tilde{h}}^{\left(l+1\right)}(v)),\;l=0,\ldots ,M-1$$ (18)

where the function *h*^(*l*)^ represents an embedding function from the *lth* layer, *u* and *v* are independent random variables that have the same probability measure *P*. The embedding functions of two consecutive layers are correlated by convolution and expressed by an integral transforma, where the kernel $\hat{A}(v,u)$ corresponds to the (*v*, *u*) element of the matrix $\hat{A}$. The loss *L* is the expected value of *g*(*h*^(*M*)^) that is finally embedded in *h*^(*M*)^, and can be expressed as:

$$L=E_{v\sim P}[g(h^{\left(M\right)})(v)]=\int g(h^{\left(M\right)})(v)dP(v)$$ (19)

For the lth layer, the t_1_ i.i.d. sample ${\text{u}}_{1}^{\left(\text{l}\right)},\ldots ,{\text{u}}_{{\text{t}}_{1}}^{\left(\text{l}\right)}\sim \text{P}$ is used to approximatively estimate the integral transformation:

$${\tilde{h}}_{t_{l+1}}^{\left(l+1\right)}(v)≔\frac{1}{t}\sum_{j=1}^{t_{l}}\hat{A}(v,u_{j}^{\left(l\right)})h_{t_{l}}^{\left(l\right)}(u_{j}^{\left(l\right)})W^{\left(l\right)},\;h_{t_{l+1}}^{\left(l+1\right)}(v)≔\sigma ({\tilde{h}}_{t_{l+1}}^{\left(l+1\right)}(v)),\;\text{l}=0,\ldots ,\text{M}-1$$ (20)

Here, $h_{t_{0}}^{\left(0\right)}$ is *h*^(0)^. Therefore, the loss *L* is transformed into:

$$L_{t_{0},t_{1},\ldots ,t_{M}}≔\frac{1}{t_{M}}\sum_{i=1}^{t_{M}}g(h_{t_{M}}^{\left(M\right)}(u_{i}^{\left(M\right)}))$$ (21)

### Prediction by forest PA classifier

In the experiment, we send the extracted features into the Forest by Penalizing Attributes (Forest PA) classifier for classification, so as to obtain accurate circRNA-disease association prediction results. Forest PA is a novel decision forest building algorithm recently proposed by Adnan *et al*. [45]. The Forest PA algorithm uses the complete attribute set to generate decision trees by imposing penalties on attributes participating in the latest decision tree. Besides, the participating attributes obtain random weights from the range of weights associated with the respective levels in the tree, thereby maintaining the decision tree generated by the algorithm with individually accuracy and diversity. The execution steps of the Forest PA algorithm are as follows:

1. The Forest PA first generates a bootstrap sample *D*_*i*_ from the original training data set *D*.
2. The Forest PA then uses the weight of attributes to generate decision trees from the bootstrap sample. When choosing the splitting attributes, Forest PA uses the CART algorithm with merit values, whose value is obtained by multiplying its classification ability with its weight.
3. The incremental values of attribute weights and gradient weight in the latest tree are updated iteratively. Here, the weights of the attributes appear in the latest tree will be updated. The weights of attributes that do not appear in the latest tree remain unchanged. Considering that the weight of attribute is determined by the level *λ* of test attributes in the latest tree, if an attribute appears on the root node, their value of *λ* is 1; if an attribute appears on the child node, their value of *λ* is 2. According to the value of *λ*, the weight of randomly generated attributes within a Weight-Range *WR* is defined as follows:
  $$WR^{\lambda }=\{\begin{array}{cc} {}[0.0, & e^{-\;\frac{1}{\lambda }}],\;if\;\lambda =1\\ {}[e^{-\;\frac{1}{\lambda -1}}+\rho , & e^{-\;\frac{1}{\lambda }}],\;if\;\lambda >1 \end{array}$$ (22)
4. Update weights of the applicable attributes with the corresponding weight increment values that do not exist in the latest tree.

### Conclusion

In this study, we proposed a new computational method called GCNCDA to predict potential circRNA-disease associations. The method makes full use of the disease semantic similarity, disease and circRNA GIP kernel similarity, the known circRNA-disease association information, and extracts the high-level abstract features from them by deep learning FastGCN algorithm. The cross-validation results show that GCNCDA performs well on the benchmark dataset circR2Disease. In comparison with different classifier models, feature extraction algorithm models, and other state-of-the-art methods, GCNCDA has exhibited strong competitiveness. Furthermore, we also predicted new circRNA-disease associations based on known associations. As a result, 16, 15 and 17 of the top 20 candidate circRNAs with the highest prediction scores in disease including breast cancer, glioma and colorectal cancer were respectively confirmed by relevant literature and databases. These experimental results indicate that GCNCDA is an effective method for predicting circRNA-disease associations and can provide highly reliable candidates for biological experiments. In future research, we will improve the FastGCN algorithm to help the model achieve better performance.

## Supporting information

S1 Table The benchmark dataset contains 739 pairs of positive samples and 739 pairs of negative samples. (XLSX)

S2 Table Names of 661 circRNAs involved in known circRNA-disease associations obtained from CircR2Disease database. (XLSX)

S3 Table Names of 100 diseases involved in known circRNA-disease associations obtained from CircR2Disease database. (XLSX)

S4 Table The MeSH dataset that provides rigorous disease classification information. (XLSX)
